# Near peer teaching in medical curricula: integrating student teachers in pathology tutorials

**DOI:** 10.3402/meo.v20.27921

**Published:** 2015-06-30

**Authors:** Nicholas Tayler, Samuel Hall, Norman J. Carr, Jonny R. Stephens, Scott Border

**Affiliations:** 1 Centre For Learning Anatomical Sciences, University Hospital Southampton, Southampton, UK; 2 Department of Pathology, University Hospital Southampton, Southampton, UK

**Keywords:** undergraduate education, peer assisted learning, social congruence, cognitive congruence

## Abstract

**Introduction:**

Due to increased cognitive and social congruence with their tutees, near peer teachers (NPTs) may be capable of more effectively delivering course material. This study examines NPTs as pathology tutors alongside more traditional teachers (e.g., consultants and registrars) to explore their acceptability, effectiveness, and years of ‘distance’ between tutors and tutees.

**Method:**

In total, 240 first- and second-year undergraduate medical students were taught set material in a pathology tutorial setting by NPTs (fourth-year medical students), registrars, or consultants. Learners were then asked to provide feedback using a 15-item, Likert-type scale.

**Results:**

On 11 of the 15 items, there were no significant differences in students’ median ratings. However, NPTs were perceived to be significantly more approachable than consultants, more aware of learning outcomes, more receptive to student input, and more invested in exam success. Compared with second-year students, first-year students showed a preference towards registrar tutors in terms of perceived gain of knowledge and use of time. In contrast, second-year students showed a preference towards NPTs, who provided more perceived knowledge gain and investment in exam success. No significant differences were found regarding consultant tutors.

**Discussion:**

Perhaps due to increased congruence with tutees, NPTs show promise as tutors within medical curricula. This provides advantages not only to tutees, but also to tutors – who may gain vital teaching experience and offer an effective supplement to ‘traditional’ faculty educators.

Peer teaching, the passage of knowledge between persons at the same or similar stage of academic training, is a long-established method of learning (1). A close variant of this is the near peer tutor (NPT), who is typically 2–5 years ahead of the tutee in learning (2). Since NPTs have usually taken the same courses and/or studied the same material, their comparable insights and experience allow for a greater cognitive (3) and social congruence (4) with other learners. Indeed, previous work in medical education has shown that the ‘distance’ between learners and NPTs is important (1). Hall et al. (5) showed that varying this distance altered the tutor–tutee dynamic and that a gap of 2–3 years between tutor and tutee was more effective than 4 years during anatomy demonstrations.

Here, we examine the role of the near peer tutor in core pathology teaching sessions, as opposed to more practical subject matter taught in an extra-curricular learning environment (1, 2, 6). In so doing, we hope to evaluate the acceptability of NPTs in curricular pathology tutorials and to identify an optimal training ‘distance’ between tutors and tutees.

## Methods

Throughout one academic year, 487 first-year and second-year medical students were taught in the undergraduate pathology tutorials at the University of Southampton. Of the 240 (49.7%) students who agreed to participate in our study, 34 were taught by a consultant pathologist, 87 by a pathology specialist registrar, and 119 by NPTs (fourth-year medical students). Near peer tutors were either volunteers or recommended to the faculty from previous NPT tutors.

All tutors were given identical material to deliver to groups of 10–17 students via any methods they desired. There were nine tutorials for first-year method students – incorporating the basics of pathology followed by tutorials on myocardial, lung, and renal diseases. Second-year students attended seven tutorials on stroke, neurology, and reproductive pathology. At the end of the academic year, participants were asked to anonymously respond to 15 questions pertaining to both quality of course and tutee–tutor congruence (e.g., How invested did the teacher appear in your exam success? How receptive was the teacher towards your input into the session?) (see Supplementary file). All items were measured on a 5-point Likert-type scale.

Ethical approval for this study was granted by the University of Southampton Ethics committee (Ethics ID 799). Data were analysed using SPSS Version 21 (IBM, Chicago). Inferential statistics utilized non-parametric tests; however, item means were used for graphical comparison purposes. Alpha was set at <0.05.

## Results

**Fig. 1:**
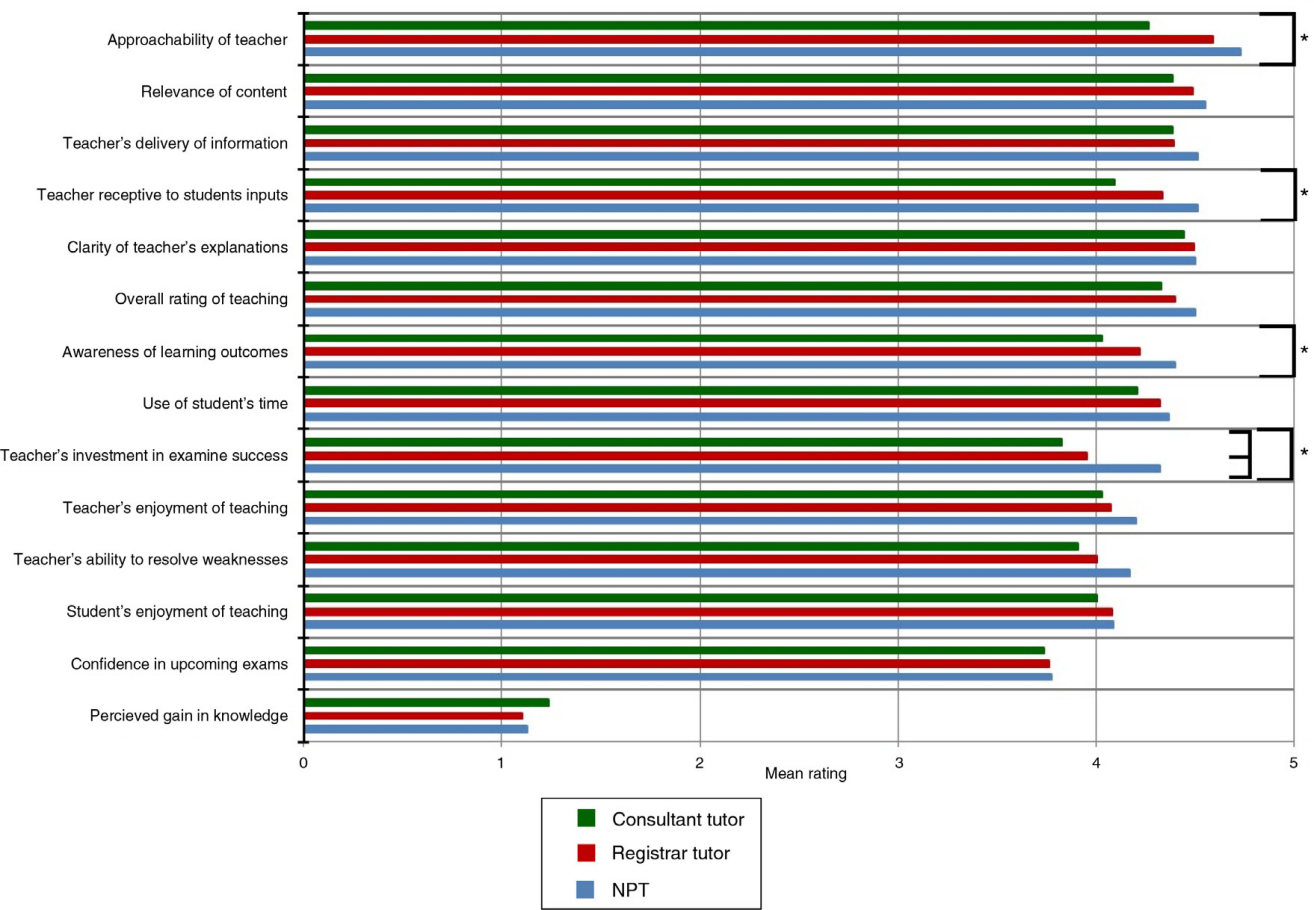
NPTs received significantly higher feedback scores than consultants in four categories (indicated by “a”) and showed no significant difference in other areas.

Of 487 students, 240 receiving teaching provided responses to our questionnaire (49.7%). No significant differences among tutor types were noted in 11 of the 15 questions (Fig. 1).

NPTs were perceived as significantly more approachable than consultant tutors (4.72 vs. 4.26), more aware of learning outcomes (4.39 vs. 4.03), and more receptive to student input (4.50 vs. 4.09). NPTs were seen as being the most invested in exam success (4.31), followed by registrars (3.95) and consultants (3.82).

When the three tutor types were compared by tutee year group, first-year students rated registrar tutors significantly higher than their second-year counterparts on the following criteria: retrospective assessment in change of perceived pre-course and post-course knowledge – knowledge gain – (1.20 vs. 0.82=24 and 16% respectively), use of time (4.42 vs. 4.04), and teacher knowledge of learning outcomes (4.34 vs. 3.86). In contrast, second-year students rated NPTs significantly higher than first-year students in knowledge gain (1.23 vs. 0.98) and tutor investment in exam success (4.49 vs. 4.01). There were no significant between-class differences in how consultants were perceived.

## Discussion

The results suggest that NPTs are equally well-accepted among students and can make excellent teachers alongside experienced subject specialists in pathology. Perhaps due to a higher level of social congruence, NPTs received higher feedback than consultants in terms of approachability. This contradicts the findings by Hall et al. (5), who found no difference between approachability when comparing senior medical student teachers with junior doctors. However, in the present study, the distance between a fourth-year medical student and registrar or consultant is much greater – particularly when student learners are in their first or second year. Whether approachability is truly a measure of congruence is also subject to some debate (5, 7).

The notion that NPTs are more aware of learning outcomes has been previously discussed. A common argument is that senior staff are too far removed from their own learning experience to know what material to focus on (8). The findings from the current study seem to support the idea that NPTs are more closely informed with the curriculum and that this is advantageous to student learners. Of course, what students may glean from NPTs’ curriculum knowledge is juxtaposed against the greater clinical experience and broader knowledge base provided by seasoned clinicians (9).

That NPTs were viewed as being most invested in exam success likely reflects the fact that they themselves had most recently experienced undergraduate examinations. Whether this represents a true advantage, or whether the focus of NPT tutorials was skewed towards passing exams, remains unexplored.

The general finding that second-year students are more accepting of NPTs is also subject to further investigation. First-year students, who themselves are new to medical training, may prefer a teacher of known (‘legitimate’) clinical or academic pedigree. However, this perception appears to change within the space of a year, and it appears that first-year students become less impressionable and/or more accepting of effective teaching regardless of the teacher's status. Further work may wish to examine this experience from the tutor's point of view.

The pathology curriculum at the University of Southampton is taught by lectures from pathologists, e-learning, and tutorials; therefore, tutors are acting more as ‘facilitators’ than ‘information providers’ (10) – which may be more suited to the NPT. The context of the teaching session may also be relevant to the apparent success of NPTs. Since all the tutors were given the same material to deliver in the same setting, decisions related to subject matter were obviously not made by the tutors themselves. It is of particular note that there was no significant difference in content relevance between tutor types.

Our results are subject to several limitations. First, since NPTs were volunteers, they may reflect students most interested in teaching and/or pathology. Second, although all tutors had access to comparable instructional technology, it is possible that more ‘traditional’ teachers were more familiar with or better-versed in its use. Finally, our use of students’ subjective, self-reported perceptions of learning would be greatly enhanced if supplemented with more objective measures of cognitive achievement.

In summary, based on the higher ratings by second-year students – and similar findings from previous studies (5) – we found the ‘distance’ along the NPT spectrum may be better suited to 2 versus 3 years. If true, the selection of NPTs may be crucial to maximizing student learning. In addition to the educational benefits to student learners, NPTs may also benefit faculty as a cost-effective supplement, and also themselves as they gain valuable teaching skills – a General Medical Council requirement (11).

## Supplementary Material

Near peer teaching in medical curricula: integrating student teachers in pathology tutorials
