# Supplementary figures and images for: Near peer teaching in medical curricula: integrating student teachers in pathology tutorials
Source: Med Educ Online. 2015 Jun 30;20:10.3402/meo.v20.27921. doi: 10.3402/meo.v20.27921 (PMC4488334; doi:10.3402/meo.v20.27921)

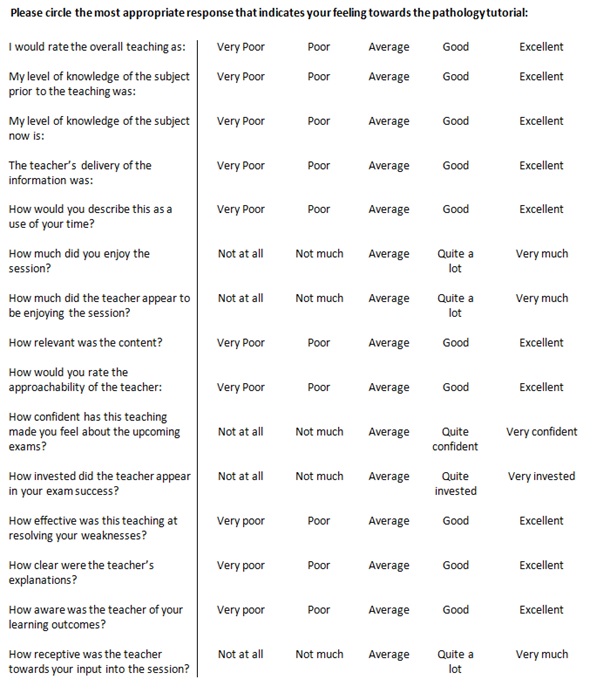

Supplement: Near peer teaching in medical curricula: integrating student teachers in pathology tutorials [file MEO-20-27921-s001.jpg]
